# Prognostic Significance of the Combined Score of Plasma Fibrinogen and Neutrophil-Lymphocyte Ratio in Patients with Spontaneous Intracerebral Hemorrhage

**DOI:** 10.1155/2021/7055101

**Published:** 2021-12-29

**Authors:** Wanchun Yang, Yunbo Yuan, Junhong Li, Yuli Shuai, Xiang Liao, Zhiyuan Yu, Hao Li, Jun Zheng

**Affiliations:** ^1^Department of Neurosurgery, West China Hospital of Sichuan University, Chengdu, 610041 Sichuan Province, China; ^2^West China School of Medicine, Sichuan University, Chengdu, 610041 Sichuan Province, China; ^3^Department of Cardiology, PLA Rocket Force Characteristic Medical Center, Beijing 100088, China

## Abstract

**Background:**

The combination of plasma fibrinogen and neutrophil to lymphocyte ratio (F-NLR) score is a novel inflammatory marker constituted by peripheral blood fibrinogen concentration and neutrophil to lymphocyte ratio. In the current study, we aim to explore the relationship between admission F-NLR score and intracerebral hemorrhage (ICH) and assess its prognostic predictive ability in ICH patients.

**Methods:**

The original cohort was consecutively recruited from August 2014 to September 2017, and the validation cohort was consecutively recruited between October 2018 and March 2020. The primary outcomes were 3-month functional outcome and 1-month mortality. All statistical analyses were performed using SPSS and R software.

**Results:**

A total of 431 and 251 ICH patients were included in original cohort and validation cohort, respectively. In the original cohort, F-NLR score could independently predict the 3-month functional outcome (adjusted OR 2.013, 95% CI 1.316-3.078, *p* = 0.001) and 1-month mortality (adjusted OR 3.036, 95% CI 1.965-4.693, *p* < 0.001). Receiver operation characteristic (ROC) analyses and predictive model comparison indicated that F-NLR score had a stronger predictive ability in the 3-month outcome and 1-month mortality. Validation cohort verified the results.

**Conclusion:**

F-NLR score was an independent indicator for both the 3-month functional outcome and 1-month mortality, and its prognostic predictive ability was superior to fibrinogen and NLR in both the original and the validation cohort.

## 1. Introduction

Nontraumatic spontaneous intracerebral hemorrhage (ICH) is the most common type of intracranial hemorrhage and isolated intraventricular hemorrhage, which accounts for 10-15% of stroke in western populations and 18-20% in Asian populations. [1, 2] ICH results from bleeding into the brain parenchymal arising from the rupture of small penetrating arteries which causes neurological deficits and death. [2]

It is well appreciated that inflammation begins immediately after the formation of hematoma and that it plays a role in ICH-induced secondary brain injury. [3, 4] Recruitment and infiltration of inflammatory cells including monocytes, macrophages, neutrophils, and lymphocytes boost local cytokines, chemokines, and adhesion molecules. [5] Accompanied with the breakdown of the blood-brain barrier (BBB), systemic inflammation also occurred. Therefore, peripheral blood inflammatory markers might also reflect the severity and progression of intracranial lesions. Studies established that neutrophil to lymphocyte ratio (NLR) was a prognostic indicator for ICH, in both early functional outcome and mortality. [6] Moreover, indexes such as monocyte, fibrinogen, albumin, C-reactive protein (CRP), and platelet to lymphocyte ratio (PLR) were also reported to be associated with the outcomes of ICH. [7–11]

The F-NLR score is a novel inflammatory marker constituted by peripheral blood fibrinogen concentration and NLR. This combined index was widely used in oncology studies and proved to have prognostic significance in various cancers including non-small cell lung cancer, atypical meningioma, and esophageal squamous cell carcinoma. [12–14] However, the evaluation of F-NLR score on spontaneous ICH was not well identified. In this study, we explored the relationship between admission F-NLR score and ICH and assessed its prognostic predictive ability in ICH patients.

## 2. Methods

### 2.1. Study Design

This current retrospective study was conducted built upon the prospectively collected database of ICH patients at the Department of Neurosurgery affiliated to West China Hospital, Sichuan University. The original cohort was consecutively recruited from August 2014 to September 2017. The validation cohort was built to verify the results from the original cohort, who were consecutively recruited patients between October 2018 and March 2020. All the patients from current cohorts were managed based on the standard guidelines for stroke. [15, 16] The baseline clinical data of the included patients were retrieved from an electronic medical record system of West China Hospital.

The inclusion criteria for screening patients included the following: (1) older than 18 years; (2) intact baseline clinical data; (3) intact imaging data including initial computed tomography (CT) at admission and computed tomography angiography (CTA); and (4) completed the follow-up. The exclusion criteria included the following: (1) younger than 18 years; (2) incomplete baseline clinical data; (3) intracerebral hemorrhage caused by head trauma, tumor, aneurysm, arteriovenous malformation, ischemic stroke, and other obvious causes; (4) absence of CTA; (5) history of infectious diseases or diagnosed with infections within 48 hours from admission, malignancies, rheumatic diseases, blood system diseases, or other diseases which evidently affect peripheral blood cells; and (6) lost to follow-up.

### 2.2. Clinical Parameters Assessment

Clinical variables were retrieved from the electronic medical record system we mentioned previously. [17] The included clinical data were listed as follows: (1) demographics: age at onset, gender; (2) admission conditions: Glasgow Coma Scale (GCS), admission systolic blood pressure (SBP), diastolic blood pressure (DBP), and time from onset to CT; (3) medical history: history of hypertension, diabetes mellitus, smoking, alcohol abuse, presence of stroke, and history of receiving antiplatelet medication; (4) ICH imaging characteristics: hematoma volume, location of hematoma, presence of intraventricular hemorrhage (IVH), and hematoma expansion (HE); (5) treatment method; and (6) routine blood test. Of note, the majority of the blood samplings were conducted when patients were diagnosed with ICH, usually after an initial CT scan.

NLR was defined as neutrophil count/lymphocyte count. Plasma fibrinogen concentration and NLR were divided into high and low groups based on the best cutoff values calculated by the receiver operating characteristic (ROC) curve. F-NLR score was defined as follows: score 0: low fibrinogen and low NLR; score 1: low fibrinogen and high NLR or high fibrinogen and low NLR; and score 2: high fibrinogen and high NLR.

The included patients were followed up every 1 month after admission. The primary outcomes were 3-month functional outcome and 1-month mortality. The modified Rankin Scale (mRS) was applied to assess the patients' functional outcome. mRS score 0-2 was defined as good outcome, and mRS score 3-6 as poor outcome. Intracranial parenchymal hematoma volume was calculated on the admission CT scans by using 3D Slicer (http://www.slicer.org) with manual segmentation. Two independent neurosurgeons were responsible for calculating hematoma volume, and a neuroradiologist was invited to advise in complex cases. Hematoma expansion was defined as hematoma enlargement ≥ 6 mL or ≥33% from admission. [18] Surgical interventions mainly consisted of hematoma evacuation with craniotomy, external ventricular drainage, and decompressive craniectomy.

### 2.3. Statistical Analysis

All statistical analysis was performed using SPSS software (Version 22.0, IBM Co., Armonk, NY, USA) and R software (Version 3.6.1). Continuous variables were displayed as mean ± standard deviation (SD) or median with interquartile range (IQR), and categorical variables were presented as frequency and percentage. A Kolmogorov–Smirnov test was applied to check whether the data conforms to the normal distribution. Continuous variables which conformed to the normal distribution were compared using student's *t*-test, otherwise Mann-Whitney U test or Kruskal-Wallis test. Categorical variables were compared using the *χ*^2^ or Fisher's exact test. The multivariate logistic regression analyses were used to confirm the independent risk factors for primary outcomes in ICH patients, and variables with *p* < 0.1 in a univariate analysis were included into backward stepwise multivariate logistic regression. ROC analyses were conducted to determine the optimal cutoff values for fibrinogen concentration and NLR by calculating maximal Youden index (Youden index = sensitivity + specificity–1) and assess the predictive accuracy of fibrinogen, NLR, and F-NLR score for outcomes. Area under the curves (AUCs) were compared by using DeLong's test. Harrell's concordance index (C-index) and Akaike information criterion (AIC) were used to evaluated the predictive models; higher C-index indicated better predictive accuracy, while lower AICs indicated superior model-fitting. [19, 20] A two-sided *p* < 0.05 was considered as statistically significant.

### 2.4. Ethics

This study was approved by the Ethical Committee of Sichuan University (No. 2013NO52) and conducted based on the principles announced in the Declaration of Helsinki. All patients and their authorized trustees were fully informed and signed consent to use their data for research.

## 3. Results

### 3.1. Baseline Clinical Characteristics

**Figure 1:**
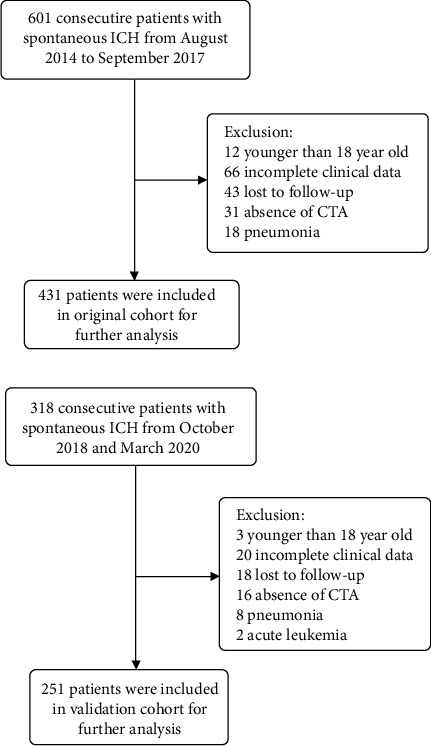
Flow chart of the current study. Abbreviation: ICH, intracerebral hemorrhage; CT, computed tomography; CTA, computed tomography angiography.

After screening, a total of 431 and 251 ICH patients were included in original cohort and validation cohort, respectively, (Figure 1). The optimal cutoff values of fibrinogen and NLR were 2.76 g/L and 10.04, respectively, in the original cohort and 2.82 g/L and 9.32, respectively, in the validation cohort by calculating the maximal Youden index through the ROC curve in the original cohort.

Baseline clinical characteristics of the original cohort are depicted in Table 1. There were 327 patients had poor outcome and 104 patients had good outcome after 3-month follow-up. As regard to 1-month mortality, 80 patients died within 30 days after admission while 351 survived. 164 (38.1%) patients had a F-NLR score 0, 183 (42.5%) had a score 1, and 84 (19.5%) had a score 2. GCS score (*p* < 0.001), smoking (*p* = 0.042), hematoma volume (*p* < 0.001), hematoma location (*p* < 0.001), IVH (*p* < 0.001), HE (*p* = 0.004), treatment method (*p* < 0.001), NLR (*p* = 0.001), fibrinogen (*p* < 0.001), and F-NLR score (*p* < 0.001) were significantly related to 3-month functional outcome. Lower GCS score (*p* < 0.001), larger hematoma volume (*p* < 0.001), presence of IVH (*p* < 0.001), undergoing surgical intervention (*p* = 0.013), higher NLR (*p* < 0.001), fibrinogen (*p* < 0.001), and F-NLR score (*p* < 0.001) associated with a higher proportion of death.

The optimal cutoff values of fibrinogen concentration and NLR were applied for validation cohort in primary outcomes. As shown in Table 2, validation cohort consisted of 179 patients with poor 3-month functional outcome and 72 with good outcome. Meanwhile, 37 patients died within 1 month. Patients with F-NLR score 0, 1, 2 were 111 (44.2%), 87 (34.7%), 53 (21.1%), respectively. GCS score (*p* < 0.001), hematoma volume (*p* < 0.001), hematoma location (*p* < 0.001), presence of IVH (*p* < 0.001), treatment method (*p* < 0.001), NLR (*p* = 0.001), fibrinogen (*p* < 0.001), and F-NLR score (*p* < 0.001) were evidently associated with 3-month functional outcome. As for 1-month mortality in validation cohort, GCS (*p* < 0.001), hematoma volume (*p* < 0.001), presence of IVH (*p* < 0.001) and HE (*p* = 0.002), platelet count (PLT) (*p* = 0.036), prothrombin time (PT) (*p* = 0.006), international normalized ratio (INR) (*p* = 0.018), NLR (*p* < 0.001), fibrinogen (*p* < 0.001), and F-NLR score (*p* < 0.001) were risk factors in univariate analysis.

### 3.2. Associations of F-NLR Score with Primary Outcomes

Multivariate logistic regression was conducted to further determine the independent risk factors for primary outcomes. In the original cohort (Table 3), F-NLR score (adjusted OR 2.013, 95% CI 1.316-3.078, *p* = 0.001; adjusted OR3.036, 95% CI1.965-4.693, *p* < 0.001, respectively) and its component fibrinogen (adjusted OR 1.852, 95% CI1.271-2.699, *p* = 0.001; adjusted OR1.946, 95% CI1.477-2.564, *p* < 0.001, respectively) could independently predict 3-month functional outcome and 1-month mortality. Whereas NLR could independently predicted 1-moth mortality (adjusted OR, 1.069, 95% CI 1.028-1.110, *p* = 0.001), but not 3-month functional outcome (adjusted OR, 1.034, 95% CI 0.981-1.089, *p* = 0.209). Similarly in validation cohort, NLR, fibrinogen, and F-NLR score were independent risk factors for primary outcomes. Other independent risk factors for ICH were listed in Supplementary Table 1 and Table 2.

### 3.3. Predictive Ability of F-NLR Score, Fibrinogen, and NLR in Primary Outcomes

**Figure 2:**
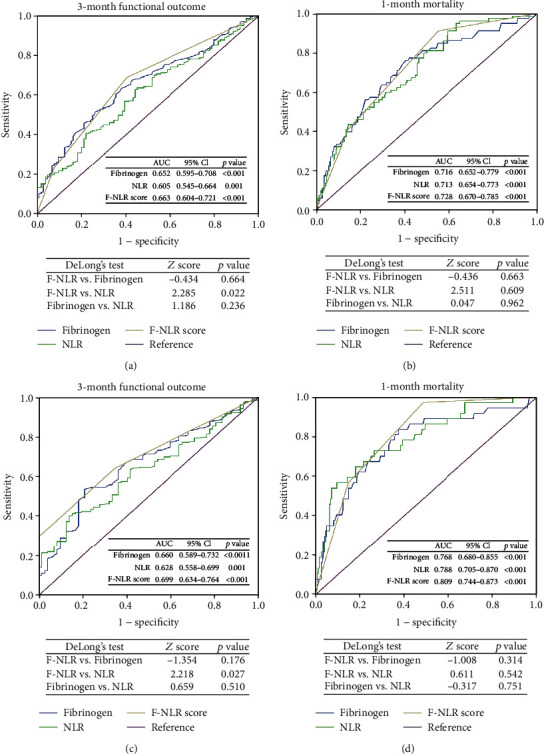
ROC curves of F-NLR score, fibrinogen, and NLR for predicting 3-month functional outcome and 1-month mortality in the original cohort (a, b) and the validation cohort (c, d). Abbreviation: ROC, receiver operating characteristic; F, fibrinogen; NLR, neutrophil to lymphocyte ratio; AUC, area under the curve; CI, confidence interval.

ROC curves were used to preliminarily assess the predictive ability of F-NLR score, fibrinogen, and NLR in primary outcomes of ICH patients. In original cohort, F-NLR score had stronger predictive ability in 3-month outcome (Figure 2(a), area under the curve (AUC) 0.663, 0.652, 0.605, respectively) and 1-month mortality (Figure 2(b), AUC 0.728, 0.716, 0.713, respectively) than fibrinogen and NLR. In validation cohort, the predictive ability in 3-month mortality (Figure 2(c), AUC 0.699, 0.660, 0.628, respectively) and 1-month mortality (Figure 2(d), AUC 0.809, 0.768, 0.788, respectively) of F-NLR score was still superior to fibrinogen and NLR. DeLong's test only indicated a significant difference between F-NLR score and NLR of AUC in 3-month functional outcome in original cohort (*p* = 0.022) and validation cohort (*p* = 0.027), whereas there was no significant difference between F-NLR score and NLR in predicting 1-month mortality. DeLong's test also indicated that no significant difference was found in AUC between fibrinogen and either of the other two markers.

Predictive models were conducted to further assess the predive accuracy of inflammatory markers in outcomes. Independent predictive indicators in multivariate logistic regression constituted predictive models. Apart from fibrinogen, NLR, and F-NLR score, other independent variables constituted basic models. As shown in Table 4, basic models plus F-NLR score had largest C-index and lowest AIC in predicting outcomes in original and validation cohorts.

## 4. Discussion

This study was conducted to explore the clinical significance of F-NLR score in ICH patients. We found that F-NLR score was an independent indicator for both the 3-month functional outcome and the 1-month mortality in original cohort, and its prognostic predictive ability was superior to fibrinogen and NLR. These results were verified in independent validation cohort.

NLR was a common peripheral marker used in fields of various disease like tumors, rheumatic diseases, cardiovascular diseases, and other chronic diseases, including corona virus disease-19 (COVID-19). [21–26] Because inflammation was characterized by higher neutrophil count and lower lymphocyte count, NLR could predict disease progression by reflecting the inflammatory burden. Based on these facts, NLR was reported to be a prognostic indicator for patients with ischemic stroke, ICH, and subarachnoid hemorrhage and also predicted the presence of stroke-associated complications, suggesting that both ischemic and hemorrhagic stroke may share common pathways. [27–33] However, controversial results showed that although inflammatory biomarkers in the serum were widely studied to predict the outcome of ICH, NLR seems to be not well enough in the prediction [34].

Fibrinogen, synthesized by hepatocytes in the liver and circulating in the bloodstream, plays a complex role in ICH. It participates in the process of blood coagulation and induces neuroinflammation by activating microglia and promoting the recruitment and activation of peripheral inflammatory macrophages into the central nervous system by converting into fibrin. [35] Inhibition of fibrin formation could reduce neuroinflammation and improve long-term outcome in mice with ICH models. [36] Studies showed that low fibrinogen concentration was an independent predictors of good outcome in medium to large spontaneous ICH. [8] Moreover, D-dimer, a soluble fibrin degradation product that results from ordered breakdown of thrombi by the fibrinolytic system, was also reported to be associated with poor outcome in ICH. [37–39]

In this study, we demonstrate that NLR combined with fibrinogen was reliable to predict the outcome of intracerebral hemorrhage than NLR or fibrinogen alone. To combine fibrinogen and NLR together, Arigami et al. firstly introduced F-NLR score in the research of esophageal squamous cell carcinoma and demonstrated that F-NLR was an independent prognostic indicator. [40] Although several oncology studies tested the prognostic significance of F-NLR score, few of them compared the predictive ability between F-NLR score with its components. Therefore, whether F-NLR score has superiority in prognostic predictive capacity is yet to be determined. It's noted that Chen et al. found F-NLR score had stronger predictive ability in 3-year progression-free survival both in training set and validation set. [13] Here, we demonstrated that F-NLR score had better predictive accuracy in primary outcomes than fibrinogen or NLR alone through ROC analysis and conducting predictive models. This easily-acquired and almost undamaged peripheral blood marker not only is economical, but also helps clinical workers distinguish the severity of ICH through combining with crucial prognostic indicators like ICH volume, presence of HE or IVH, thus undertaking inchoate interventions and making the optimal treatment plans. Combined with imaging characteristic like spot sign, island sign and satellite sign, the prognostic models will be enriched to further improve the predictive accuracy in disease-progression and prognosis of ICH. [41] In addition, a variety of factors related to metabolic homeostasis, blood perfusion, drug reaction, and condition of nutrition are strongly affecting the prognosis in short and long-term outcomes, multidimensional and comprehensive evaluations of ICH patients are needed. [42–46] There were still some limitations that merit consideration. First, current results were concluded from a single center, and multicenter collaboration will be needed for further verification. Second, not all patients were admitted to hospital within similar intervals after onset, this might induce unknown bias in laboratory results. Third, severe cases with early mortality after admission might cause selection bias. Finally, long-term outcomes should be introduced to comprehensively evaluate the prognostic significance of candidate markers in the future.

## 5. Conclusion

In summary, we demonstrate that F-NLR score was an independent indicator for both 3-month functional outcome and 1-month mortality, and its prognostic predictive ability was superior to fibrinogen and NLR in both the original and validation cohorts. Our findings provide a novel prognostic predictor for the outcome of ICH and deserve potential clinical application.

## Figures and Tables

**Table 1:** Baseline clinical characteristics of patients with spontaneous ICH in original cohort.

Clinical variables	Total (*n* = 431)	3-month functional outcome			1-month mortality		
		Poor outcome (*n* = 327)	Good outcome (*n* = 104)	*p* value	Death (*n* = 80)	Survival (*n* = 351)	*p* value
Age (year)	58.76 ± 12.92	59.09 ± 13.10	57.72 ± 12.34	0.557	58.88 ± 13.77	58.74 ± 12.74	0.981
Male	299 (69.4)	227 (69.4)	72 (69.2)	0.971	53 (66.3)	246 (70.1)	0.502
GCS	13 (8,15)	12 (7,14)	15 (14,15)	*<0.001*	8 (6,11)	13 (10,15)	*<0.001*
Admission SBP (mmHg)	167.72 ± 28.65	168.77 ± 29.76	164.42 ± 24.68	0.117	164.30 ± 31.79	168.50 ± 27.88	0.498
Admission DBP (mmHg)	98.10 ± 17.94	98.08 ± 18.46	98.16 ± 16.31	0.723	95.04 ± 19.41	98.79 ± 17.55	0.114
Onset to CT (hours)	6 (4,12)	6 (4,10)	7 (4,20)	0.020	5 (4,9)	6 (4,13)	0.262
History of hypertension	245 (56.8)	188 (57.5)	57 (54.8)	0.630	46 (57.5)	199 (56.7)	0.896
History of diabetes mellitus	31 (7.2)	28 (8.6)	3 (2.9)	0.051	3 (3.8)	28 (8.0)	0.187
Smoking	143 (33.2)	117 (35.8)	26 (25.0)	*0.042*	23 (28.7)	120 (34.2)	0.351
Previous stroke	22 (5.1)	19 (5.8)	3 (2.9)	0.238	2 (2.5)	20 (5.7)	0.373
Antiplatelet or anticoagulation	20 (4.6)	14 (4.3)	6 (5.8)	0.718	5 (6.3)	15 (4.3)	0.643
Hematoma volume (mL)	20.30 (7.81,35.78)	25.16 (9.06,40.42)	9.25 (2.64,21.70)	*<0.001*	28.69 (10.72,49.69)	17.69 (7.05,32.94)	*<0.001*
Hematoma location							
Supratentorial	358 (83.1)	284 (86.9)	74 (71.2)	*<0.001*	66 (82.5)	292 (83.2)	0.882
Infratentorial	73 (16.9)	43 (13.1)	30 (28.8)		14 (17.5)	59 (16.8)	
Presence of IVH	170 (39.4)	147 (45.0)	23 (22.1)	*<0.001*	57 (71.3)	113 (32.2)	*<0.001*
Presence of HE	104 (24.1)	90 (27.5)	14 (13.5)	*0.004*	25 (31.3)	79 (22.5)	0.099
Treatment							
Surgical intervention	114 (26.5)	107 (32.7)	7 (6.7)	*<0.001*	30 (37.5)	84 (23.9)	*0.013*
Conservative treatment	317 (73.5)	220 (67.3)	97 (93.3)		50 (62.5)	267 (76.1)	
PLT (10ˆ9/L)	151 (115,199)	151 (114,198)	150 (120,205)	0.824	143 (112,199)	151 (116,201)	0.374
PT (s)	11.4 (10.9,12.3)	11.4 (10.9,12.3)	11.6 (10.9,12.3)	0.482	11.8 (10.9,13.0)	11.4 (10.9,12.1)	0.075
APTT (s)	26.5 (23.7,29.7)	26.5 (23.7,29.4)	26.6 (23.8,30.3)	0.811	26.5 (23.5,30.1)	26.6 (23.8,29.5)	0.974
INR	0.99 (0.93-1.06)	0.98 (0.93,1.05)	1.00 (0.94,1.06)	0.153	1.00 (0.93,1.10)	0.98 (0.93,1.04)	0.066
NLR	8.18 (4.81,13.20)	8.77 (5.14,13.88)	6.44 (4.25,10.49)	*0.001*	12.53 (7.99,19.48)	7.35 (4.32,11.98)	*<0.001*
Fibrinogen	2.76 (2.29,3.38)	2.92 (2.37,3.54)	2.44 (2.20,2.97)	*<0.001*	3.34 (2.87,4.47)	2.65 (2.23,3.23)	*<0.001*
F-NLR score							
0	164 (38.1)	102 (31.2)	62 (59.6)	*<0.001*	7 (8.8)	157 (44.7)	*<0.001*
1	183 (42.5)	149 (45.6)	34 (32.7)		40 (50.0)	143 (40.7)	
2	84 (19.5)	76 (23.2)	8 (7.7)		33 (41.3)	51 (14.5)	

Data are expressed as *n* (%), mean ± SD, or median (25^th^, 75^th^ quartile). Abbreviation: ICH, intracerebral hemorrhage; SD, standard deviation; GCS, Glasgow Coma Scale; SBP, systolic blood pressure; DBP, diastolic blood pressure; IVH, intraventricular hemorrhage; HE, hematoma expansion; PLT, platelet; PT, prothrombin time; APTT, activated partial thromboplastin time; INR, international normalized ratio; F, fibrinogen; NLR, neutrophil to lymphocyte ratio.

**Table 2:** Baseline clinical characteristics of patients with spontaneous ICH in validation cohort.

Clinical variables	Total (*n* = 251)	3-month functional outcome			1-month mortality		
		Poor outcome (*n* = 179)	Good outcome (*n* = 72)	*p* value	Death (*n* = 37)	Survival (*n* = 214)	*p* value
Age (year)	59.21 ± 13.57	59.88 ± 13.78	57.56 ± 13.00	0.561	58.76 ± 14.16	59.29 ± 13.50	0.584
Male	166(66.1)	113(63.1)	53(73.6)	0.112	23(62.2)	143(66.8)	0.580
GCS	14(10,15)	13(8,15)	15(14,15)	*<0.001*	8(7,13)	14(12,15)	*<0.001*
Admission SBP (mmHg)	166.69 ± 26.58	167.77 ± 26.72	164.03 ± 26.23	0.342	160.14 ± 25.42	167.83 ± 26.67	0.128
Admission DBP (mmHg)	97.59 ± 18.51	97.47 ± 18.31	97.86 ± 19.12	0.833	93.73 ± 18.31	98.25 ± 18.50	0.155
Onset to CT (hours)	6(4,12)	6(4,12)	6(4,17)	0.375	6(3,17)	6(4,12)	0.854
History of hypertension	142(56.6)	104(58.1)	38(52.8)	0.342	18(48.6)	124(57.9)	0.292
History of diabetes mellitus	16(6.4)	14(7.8)	2(2.8)	0.233	0(0.0)	16(7.5)	0.176
Smoking	71(28.3)	54(30.2)	17(23.6)	0.297	6(16.2)	65(30.4)	0.077
Previous stroke	13(5.2)	10(5.6)	3(4.2)	0.885	0(0.0)	13(6.1)	0.255
Antiplatelet or anticoagulation	19(7.6)	11(6.1)	8(11.1)	0.179	4(10.8)	15(7.0)	0.638
Hematoma volume (mL)	18.48(7.80,35.20)	22.80(9.24,36.75)	9.22(2.66,27.27)	*<0.001*	35.34(20.70,48.78)	15.72(7.01,31.49)	*<0.001*
Hematoma location							
Supratentorial	207(82.5)	159(88.8)	48(66.7)	*<0.001*	33(89.2)	174(82.2)	0.244
Infratentorial	44(17.5)	20(11.2)	24(33.3)		4(10.8)	40(17.8)	
Presence of IVH	91(36.3)	80(44.7)	11(15.3)	*<0.001*	24(64.9)	67(31.3)	*<0.001*
Presence of HE	59(23.5)	47(26.3)	12(16.7)	0.105	16(43.2)	43(20.1)	*0.002*
Treatment							
Surgical intervention	49(19.5)	45(25.1)	4(5.6)	*<0.001*	11(29.7)	38(17.8)	0.090
Conservative treatment	202(80.5)	134(74.9)	68(94.4)		26(70.3)	176(82.2)	
PLT (10ˆ9/L)	152(115,204)	152(114,203)	153(116,210)	0.634	127(105,195)	157(120,209)	*0.036*
PT (s)	11.5(10.9,12.4)	11.4(10.9,12.2)	11.5(10.9,12.5)	0.659	12.4(11.1,13.2)	11.4(10.9,12.1)	*0.006*
APTT (s)	26.5(23.6,29.3)	26.3(23.6,29.0)	26.7(23.4,29.8)	0.634	27.8(24.8,31.4)	26.3(23.6,29.0)	0.069
INR	0.99(0.93,1.06)	0.98(0.93,1.06)	1.00(0.94,1.08)	0.254	1.05(0.94,1.13)	0.98(0.93,1.05)	*0.018*
NLR	8.18(4.88,12.72)	8.94(5.38,13.88)	6.45(4.48,9.73)	*0.001*	16.73(9.05,21.89)	7.27(4.48,10.79)	*<0.001*
Fibrinogen (g/L)	2.76(2.37,3.34)	2.98(2.43,3.51)	2.50(2.25,2.87)	*<0.001*	3.54(3.04,4.82)	2.65(2.32,3.22)	*<0.001*
F-NLR score							
0	111(44.2)	64(35.8)	47(65.3)	*<0.001*	1(2.7)	110(51.4)	*<0.001*
1	87(34.7)	62(34.6)	25(34.7)		15(40.5)	72(33.6)	
2	53(21.1)	53(29.6)	0(0.0)		21(56.8)	32(15.0)	

Data are expressed as *n* (%), mean ± SD, or median (25^th^, 75^th^ quartile). Abbreviation: ICH, intracerebral hemorrhage; SD, standard deviation; GCS, Glasgow Coma Scale; SBP, systolic blood pressure; DBP, diastolic blood pressure; IVH, intraventricular hemorrhage; HE, hematoma expansion; PLT, platelet; PT, prothrombin time; APTT, activated partial thromboplastin time; INR, international normalized ratio; F, fibrinogen; NLR, neutrophil to lymphocyte ratio.

**(a):** 

Original cohort	Multivariate logistic regression	
3-month functional outcome	Adjusted OR (95% CI)	*p* value
NLR (per 1 point increase)	1.034(0.981-1.089)	0.209
Fibrinogen (per 1 g/L increase)	1.852(1.271-2.699)	*0.001*
F-NLR score (per 1 point increase)	2.013(1.316-3.078)	*0.001*
1-month mortality	Adjusted OR (95% CI)	*p* value
NLR (per 1 point increase)	1.069(1.028-1.110)	*0.001*
Fibrinogen (per 1 g/L increase)	1.946(1.477-2.564)	*<0.001*
F-NLR score (per 1 point increase)	3.036(1.965-4.693)	*<0.001*

**(b):** 

Validation cohort	Multivariate logistic regression	
3-month functional outcome	Adjusted OR (95% CI)	*p* value
NLR (per 1 point increase)	1.086(1.015-1.163)	*0.018*
Fibrinogen (per 1 g/L increase)	1.857(1.137-3.032)	*0.013*
F-NLR score (per 1 point increase)	4.008(2.267-7.088)	*<0.001*
1-month mortality	Adjusted OR (95% CI)	*p* value
NLR (per 1 point increase)	1.181(1.099-1.268)	*<0.001*
Fibrinogen (per 1 g/L increase)	2.400(1.444-3.987)	*0.001*
F-NLR score (per 1 point increase)	7.629(3.524-16.516)	*<0.001*

Abbreviation: F, fibrinogen; NLR, neutrophil to lymphocyte ratio; OR, odds ratio; CI, confidence interval.

**(a):** 

Original cohort	3-month functional outcome		Original cohort	1-month mortality	
Predictive models	C-index	AIC	Predictive models	C-index	AIC
Basic model^§^	0.8616	339.4594	Basic model^†^	0.8203	330.1387
Basic model+fibrinogen	0.8729	331.9510	Basic model+fibrinogen	0.8518	306.0032
Basic model+NLR	0.8634	338.3470	Basic model+NLR	0.8460	318.2431
Basic model+F-NLR score	0.8731	329.4173	Basic model+F-NLR score	0.8589	306.0029

**(b):** 

Validation cohort	3-month functional outcome		Validation cohort	1-month mortality	
Predictive models	C-index	AIC	Predictive models	C-index	AIC
Basic model^‡^	0.8108	240.9239	Basic model^∗^	0.7834	179.8238
Basic model+fibrinogen	0.8319	232.0876	Basic model+fibrinogen	0.8362	162.7893
Basic model+NLR	0.8300	232.9518	Basic model+NLR	0.8591	149.2128
Basic model+F-NLR score	0.8694	209.8464	Basic model+F-NLR score	0.8876	147.3019

^§^Basic model: GCS, hematoma volume, hematoma location, treatment;^†^basic model: GCS, hematoma volume, presence of IVH, PT;^‡^Basic model: GCS, hematoma location, treatment;^∗^basic model: GCS, hematoma volume, presence of HE, PLT, PT. Abbreviation: GCS, Glasgow Coma Scale; IVH, intraventricular hemorrhage; HE, hematoma expansion; PLT, platelet; PT, prothrombin time; C-index, Harrell's concordance index; AIC, Akaike information criterion; F, fibrinogen; NLR, neutrophil to lymphocyte ratio; OR, odds ratio; CI, confidence interval.

## Data Availability

The datasets for this study are available from the corresponding author on reasonable request.

## Supplementary Materials

Supplementary Materials Supplementary Table 1. Multivariate logistic regression of included clinical variables for primary outcomes in original cohort. Supplementary Table 2 Multivariate logistic regression of included clinical variables for primary outcomes in validation cohort.
