# Supplementary material for: Prognostic Significance of the Combined Score of Plasma Fibrinogen and Neutrophil-Lymphocyte Ratio in Patients with Spontaneous Intracerebral Hemorrhage
Source: Dis Markers. 2021 Dec 29;2021:7055101. doi: 10.1155/2021/7055101 (PMC8731292; doi:10.1155/2021/7055101)
Supplement: Supplementary Materials — Supplementary Table 1. Multivariate logistic regression of included clinical variables for primary outcomes in original cohort. Supplementary Table 2 Multivariate logistic regression of included clinical variables for primary outcomes in validation cohort. [file 7055101.f1.docx]

| **Primary outcome** | Multivariate analysis | | Multivariate analysis | |
| --- | --- | --- | --- | --- |
|  | (NLR, Fibrinogen) | | (F-NLR score) | |
| **3-month functional outcome** | adjusted OR (95%CI) | p value | adjusted OR (95%CI) | p value |
| GCS (per 1 point increase) | 0.636(0.553-0.731) | ***<0.001*** | 0.638(0.556-0.733) | ***<0.001*** |
| Onset to CT (per 1 hour increase) | 0.987(0.971-1.003) | 0.114 | 0.988(0.973-1.004) | 0.15 |
| History of diabetes mellitus (Yes VS No) | 3.098(0.752-12.773) | 0.118 | 3.403(0.791-14.638) | 0.100 |
| Smoking (Yes VS No) | 1.800(0.981-3.303) | 0.058 | 1.658(0.906-3.035) | 0.101 |
| Hematoma volume (per 1ml increase) | 1.027(1.007-1.047) | ***0.008*** | 1.027(1.007-1.047) | ***0.009*** |
| Hematoma location (Supratentorial VS infratentorial) | 2.679(1.211-5.924) | ***0.015*** | 2.863(1.298-6.313) | ***0.009*** |
| Presence of IVH (Yes VS No) | 1.133(0.581-2.208) | 0.714 | 1.101(0.575-2.106) | 0.772 |
| Presence of HE (Yes VS No) | 1.014(0.459-2.241) | 0.973 | 0.907(0.415-1.981) | 0.806 |
| Treatment (Surgical intervention VS Conservative treatment) | 3.047(1.225-7.582) | ***0.017*** | 3.294(1.327,8.175) | ***0.010*** |
| NLR (per 1 point increase) | 1.034(0.981-1.089) | 0.209 |  |  |
| Fibrinogen (per 1 g/L increase) | 1.852(1.271,2.699) | ***0.001*** |  |  |
| F-NLR score (per 1 point increase) |  |  | 2.013(1.316-3.078) | ***0.001*** |
|  |  |  |  |  |
| **1-month mortality** |  |  |  |  |
| GCS (per 1 point increase) | 0.775(0.711-0.846) | ***<0.001*** | 0.769(0.706-0.837) | ***<0.001*** |
| Hematoma volume (per 1ml increase) | 1.024(1.010-1.038) | ***0.001*** | 1.022(1.009-1.036) | ***0.001*** |
| Presence of IVH (Yes VS No) | 2.365(1.237-4.521) | ***0.009*** | 2.362(1.260-4.430) | ***0.007*** |
| Presence of HE (Yes VS No) | 1.034(0.514-2.079) | 0.925 | 0.934(0.468-1.863) | 0.847 |
| Treatment (Surgical intervention VS Conservative treatment) | 1.134(0.570-2.254) | 0.720 | 1.173(0.593-2.321) | 0.646 |
| PT (per 1 second increase) | 1.135(1.025-1.257) | ***0.015*** | 1.143(1.029-1.270) | ***0.013*** |
| INR (per 1 point increase) | 1.281(0.796-2.063) | 0.307 |  |  |
| NLR (per 1 point increase) | 1.069(1.028-1.110) | ***0.001*** |  |  |
| Fibrinogen (per 1 g/L increase) | 1.946(1,477-2.564) | ***<0.001*** |  |  |
| F-NLR score (per 1 point increase) |  |  | 3.036(1.965-4.693) | ***<0.001*** |

**Supplementary Table 1. Multivariate logistic regression of included clinical variables for primary outcomes in original cohort.**

Abbreviations: GCS, Glasgow Coma Scale; IVH, intraventricular hemorrhage; HE, hematoma expansion; F, fibrinogen; NLR, neutrophil to lymphocyte ratio; OR, odds ratio; CI, confidence interval.

| **Primary outcome** | Multivariate analysis | | Multivariate analysis | |
| --- | --- | --- | --- | --- |
|  | (NLR, Fibrinogen) | | (F-NLR score) | |
| **3-month functional outcome** | adjusted OR (95%CI) | p value | adjusted OR (95%CI) | p value |
| GCS (per 1 point increase) | 0.674(0.572-0.794) | ***<0.001*** | 0.660(0.557-0.782) | ***<0.001*** |
| Hematoma volume (per 1ml increase) | 1.007(0.983-1.032) | 0.582 | 1.006(0.981-1.032) | 0.622 |
| Hematoma location (Supratentorial VS infratentorial) | 4.660(1.964-11.054) | ***<0.001*** | 4.561(1.871-11.119) | ***0.001*** |
| Presence of IVH (Yes VS No) | 1.773(0.771-4.076) | 0.178 | 1.784(0.764-4.168) | 0.181 |
| Treatment (Surgical intervention VS Conservative treatment) | 4.116(1.309-12.948) | ***0.016*** | 5.147(1.614-16.413) | ***0.006*** |
| NLR (per 1 point increase) | 1.086(1.015-1.163) | ***0.018*** |  |  |
| Fibrinogen (per 1 g/L increase) | 1.857(1.137-3.032) | ***0.013*** |  |  |
| F-NLR score (per 1 point increase) |  |  | 4.008(2.267-7.088) | ***<0.001*** |
|  |  |  |  |  |
| **1-month mortality** |  |  |  |  |
| GCS (per 1 point increase) | 0.791(0.673-0.931) | ***0.005*** | 0.783(0.676-0.909) | ***0.001*** |
| Hematoma volume (per 1ml increase) | 1.066(1.034-1.099) | ***<0.001*** | 1.053(1.026-1.081) | ***<0.001*** |
| Presence of IVH (Yes VS No) | 1.460(0.438-4.873) | 0.538 | 0.988(0.325-3.000) | 0.983 |
| Presence of HE (Yes VS No) | 3.306(1.089-10.035) | ***0.035*** | 4.102(1.406-11.966) | ***0.010*** |
| Treatment (Surgical intervention VS Conservative treatment) | 0.801(0.200-3.202) | 0.753 | 0.833(0.231-3.001) | 0.780 |
| PLT (per 1 point increase) | 0.986(0.976-0.996) | ***0.007*** | 0.988(0.979-0.997) | ***0.007*** |
| PT (per 1 second increase) | 1.216(1.085-1.363) | ***0.001*** | 1.193(1.063-1.340) | ***0.003*** |
| INR (per 1 point increase) | 1.126(0.576-2.201) | 0.728 | 1.107(0.563-2.180) | 0.768 |
| NLR (per 1 point increase) | 1.181(1.099-1.268) | ***<0.001*** |  |  |
| Fibrinogen (per 1 g/L increase) | 2.400(1.444-3.987) | ***0.001*** |  |  |
| F-NLR score (per 1 point increase) |  |  | 7.629(3.524-16.516) | ***<0.001*** |

**Supplementary Table 2. Multivariate logistic regression of included clinical variables for primary outcomes in validation cohort.**

Abbreviations: GCS, Glasgow Coma Scale; IVH, intraventricular hemorrhage; HE, hematoma expansion; PLT, platelet; PT, prothrombin time; INR, international normalized ratio; F, fibrinogen; NLR, neutrophil to lymphocyte ratio; OR, odds ratio; CI, confidence interval.
